# Supplementary material for: The interactome of intact mitochondria by cross-linking mass spectrometry provides evidence for coexisting respiratory supercomplexes
Source: Mol Cell Proteomics. 2017 Dec 8;17(2):216–32. doi: 10.1074/mcp.RA117.000470 (PMC5795388; doi:10.1074/mcp.RA117.000470)
Supplement: Supplemental Data [file supp_RA117.000470_133922_0_supp_23978_fzffwf.zip › spectra_annotation/mito_spectra_annotation/111-1-4-1-21-1.pdf]

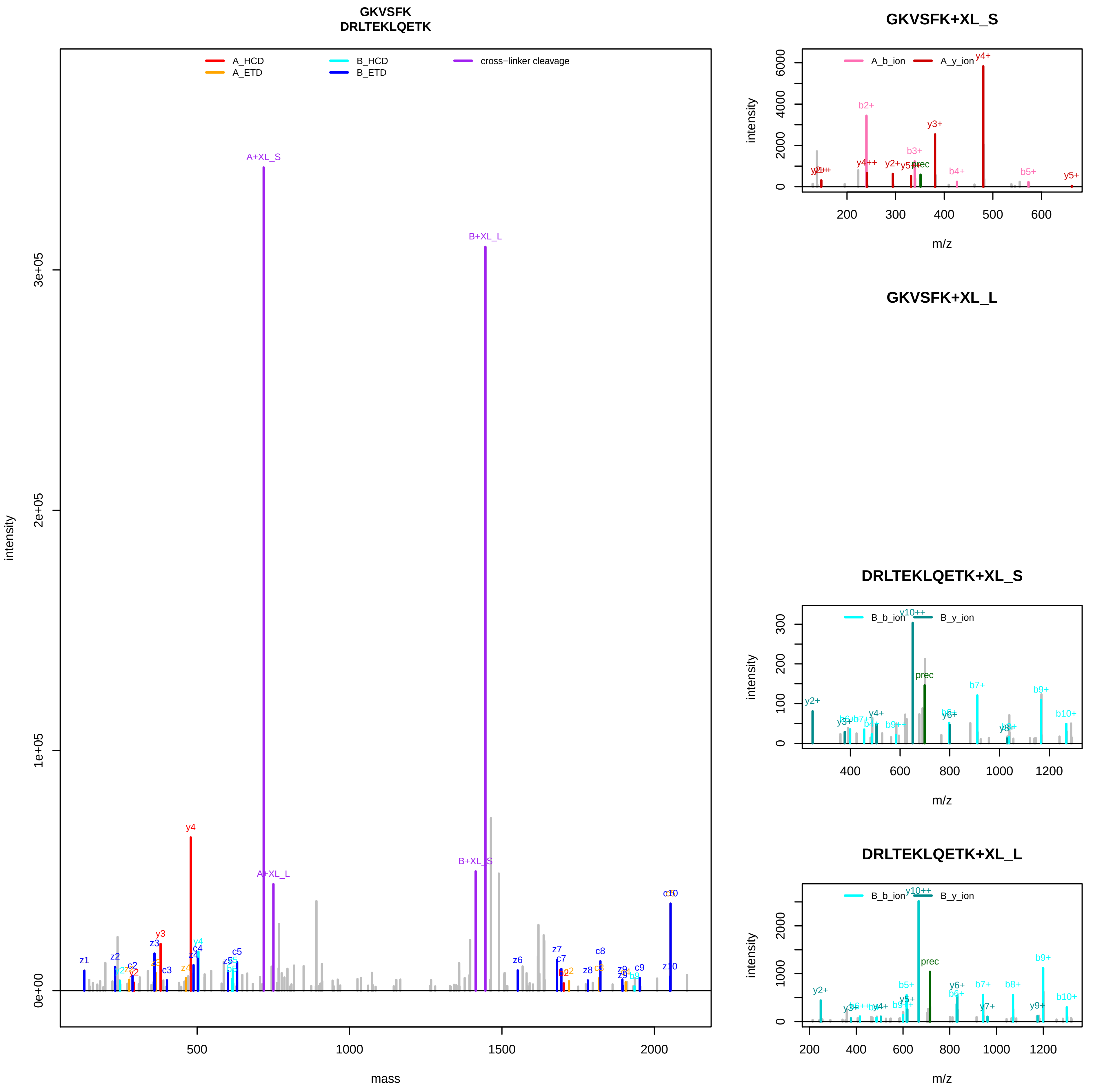
GKVSFK
DRLTEKLQETK
A_HCD
A_ETD
B_HCD
B_ETD
cross−linker cleavage
A+XL_S
B+XL_L
A+XL_L
B+XL_S
y4
y3
z1
z2
c2
z3
c3
c4
z4
c5
z5
z6
z7
c7
z8
c8
z9
c9
c10
z10
0e+00
1e+05
2e+05
3e+05
intensity
500
1000
1500
2000
mass
GKVSFK+XL_S
A_b_ion
A_y_ion
y4+
b2+
y3+
b3+
y4++
y2+
prec
b4+
b5+
y5+
0
2000
4000
6000
200
300
400
500
600
m/z
GKVSFK+XL_L
DRLTEKLQETK+XL_S
B_b_ion
B_y_ion
y10++
prec
b7+
b9+
y2+
y4+
y3+
b6+
y6+
b10+
0
100
200
300
400
600
800
1000
1200
DRLTEKLQETK+XL_L
y10++
prec
b9+
y2+
b5+
y6+
b7+
b8+
y5+
y3+
y4+
y7+
y9+
b10+
0
1000
2000
200
